# Five-year follow-up of mitral valve repair versus replacement: a propensity score analysis

**DOI:** 10.1186/s13019-023-02144-1

**Published:** 2023-01-16

**Authors:** Majd Makarious Laham, Jerry Easo, Marcin Szczechowicz, Mehdy Roosta-Azad, Alexander Weymann, Arjang Ruhparwar, Markus Kamler

**Affiliations:** West German Heart and Vascular Center, Heart Surgery Huttrop, University Hospital of Essen, Herwarth Str 100, 45138 Essen, Germany

**Keywords:** Mitral valve, Repair, Replacement, Mitral valve regurgitation

## Abstract

**Background:**

Mitral valve repair (MVRe) is considered to have a superior outcome compared to replacement (MVRp) in patients with mitral valve regurgitation (MVR). It was the aim of the study to analyse the clinical results and identify risk factors for short and long-term mortality.

**Methods:**

In a retrospective single-center analysis, patients undergoing an isolated mitral valve procedure from June 2010 to December 2016 were identified. These were subsequently homogenized using 10 baseline characteristics for propensity-score matching. Comparative analyses were performed for early and long-term results, using adequate statistical tools, and identifying risk factors for the investigated endpoints, primary end-point: all-cause mortality within 5 years and secondary end-points: recurrent MVR, reoperation, endocarditis and/or mortality with 30 days, 1, 3 and 5 years.

**Results:**

241 patients were identified in the entire patient cohort. After matching, patients were divided into 2 groups of 64 each respectively. The median age was similar in the two groups. There was a significant interaction between early mortality risk of MV in patients with coronary artery disease (CAD) (OR 11.94, 95% CI 1.49–285.92, *p* = 0.04) and late mortality in patients with higher EuroSCORE II (HR 1.14, 95% CI 1.06–1.23, *p* < 0.001). The primary end-point showed 5-year survival rate was significantly higher in MVRe versus MVRp (90.06% vs. 79.54% respectively, *p* = 0.04). The secondary end-point demonstrated recurrent MVR not to be statistically significant between the 2 groups (*p* = 0.09) as well as reoperation (*p* = 0.28). Endocarditis was observed in one patient after MVRp.

**Conclusions:**

We concluded MVRe to be associated with lower operative and 5-year mortality and good postoperative outcomes compared to patients undergoing MVRp. Concomitant CAD was identified as one of the risk factors for increasing the in-hospital mortality rate. There was no significant difference in rehospitalisation over the follow-up period. MVRe should be the treatment of choice for severe MVR and should remain a central aspect in valve centers' treatment algorithms and quality measures.

## Introduction

Severe mitral valve regurgitation (MVR) is a common valvular heart disease with an unfavorable prognosis when left untreated [1, 2], and with a prevalence of 2% in the general population and a steep increase as a function of age [3].

When studying mitral valve pathology and its surgical implications it is of necessity to assess the etiology of the disease. Acute MVR may occur due to rupture of the papillary muscle in patients with acute ST-segment elevation myocardial infarction (STEMI) [4]. It may also present due to disruption of different parts of the mitral valve apparatus via infective endocarditis or spontaneous chordal rupture in patients with degenerative mitral valve disease. When assessing patients with chronic MVR, it is critical to distinguish between chronic primary (degenerative) and chronic secondary (functional) MVR, as these conditions are unequal [4] with the subsequent implications for treatment.

Degenerative disease of the mitral valve represents 60–70% of patients undergoing surgery due to mitral regurgitation in industrialized nations [5] and is most commonly related to prolapse of the mitral valve with a spectrum of pre-existing conditions, ranging from a single prolapsing valve segment to diffuse myxomatous degeneration with bi-leaflet prolapse and annular dilatation [5, 6].

Treatment algorithms have been redefined in recent years as a result of the excellent outcomes of surgical repair with a recommendation of risk stratification and earlier intervention when the probability of durable repair is high and surgery can be undertaken by experienced teams with high repair rates and low operative mortality and morbidity rates [2, 4, 7]. Numerous studies comparing mitral valve repair to replacement have demonstrated a possible survival benefit for repair, with excellent safety and durability. However, these findings are controversial in light of reports showing a benefit for preventing recurrence of mitral valve regurgitation and rehospitalisation when undergoing mitral valve replacement, and in regard to present reports, there are no randomized studies asserting MV repair to be preferred to replacement [8]. Nevertheless, some studies emphasized the advantages of MV repair due to its lower operative mortality [9–11].

Our study aims to: (1) assess our institutional results of mitral valve surgery classifying into repair and replacement groups, (2) to compare intra and postoperative results over a time span of 9 years, (3) To identify possible risk factors influencing early and long-term results and (4) to analyse the databank to obtain information to facilitate the decision-making process in consideration of our results.

## Methods

### Study design

**Fig. 1:**
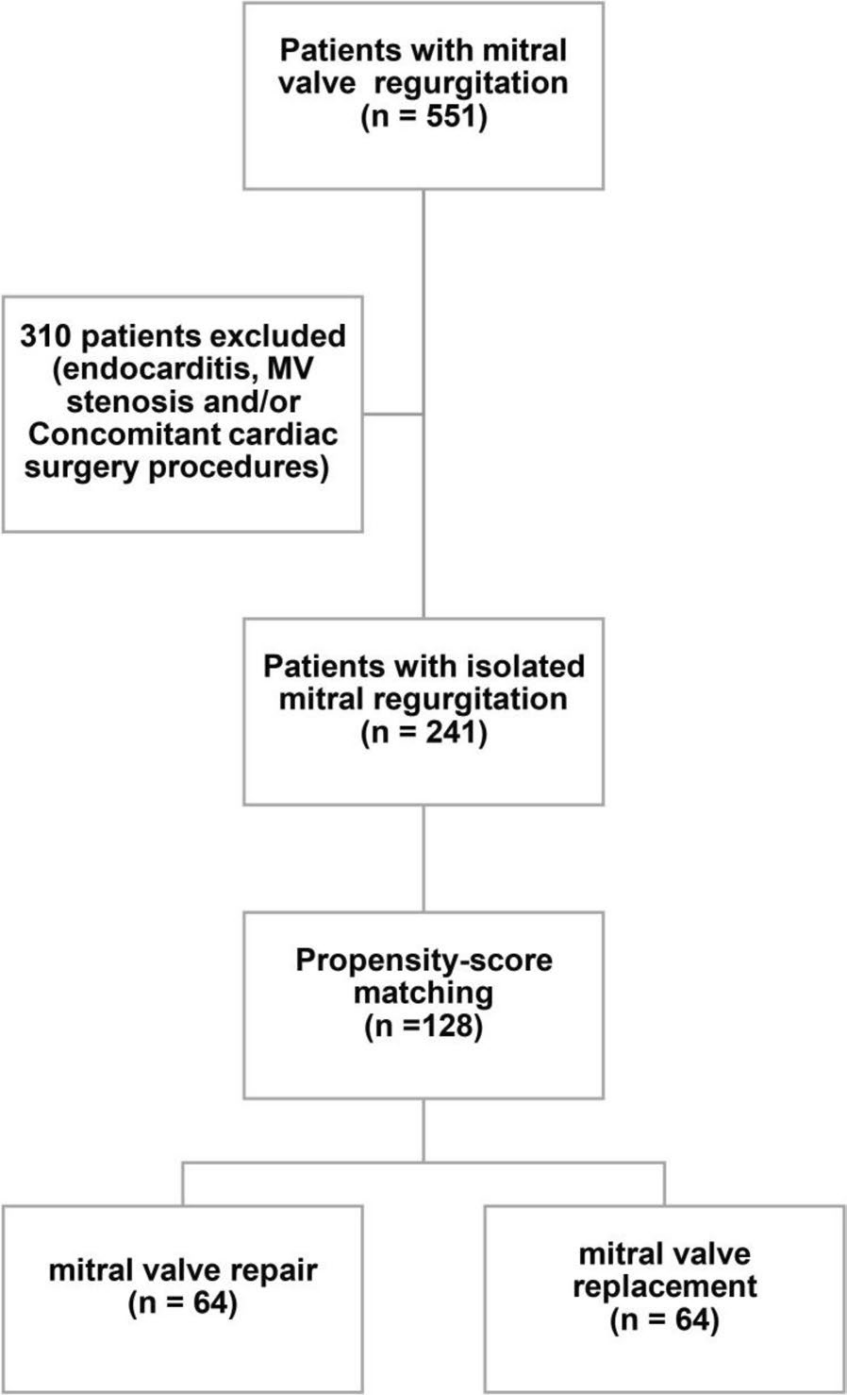
Study design

In this retrospective study, 241 patients underwent an isolated mitral valve (MV) surgery in our center between June 2010 and December 2016. Patients who had active endocarditis and/or mitral valve stenosis were excluded from our study. Propensity score matching for 10 baseline characteristics was used (Table 1). 128 patients were identified and classified into two groups; the MV repair group (MVRe) and the MV replacement group (MVRp), with 64 patients in each group respectively. The median follow-up was 5.5 years (range 0.003–9.101 years) after surgery (Fig. 1). The Indication of surgery was moderate to severe primary as well as secondary MVR, and we classified the MV pathology based on the Carpentier classification (Cc).

**Table 1:** Propensity match score characteristics

Age
Gender
History of coronary heart disease
History of atrial fibrillation
History of stroke
History of previous cardiac surgery
NYHA classification
Obesity
Atrial hypertension
Preoperative Echocardiography (left atrium diameter ≤ 50 mm)

### Surgical techniques

Mitral valve surgery was performed with the use of cardiopulmonary bypass using cold crystalloid cardioplegic arrest. Bicaval cannulation was used and exposition of the mitral valve was achieved via a bi-atrial vertical transseptal incision or via the Waterston’s incision. Using moderate hypothermia (32–34 °C) in the majority of the patients treated.

In the repair group, the mitral valve was corrected using the “respect” technique by the insertion of 2–5 artificial Gore-Tex Neo-Chordae to re-suspend the prolapsing segments in the presence of chordal rupture or elongation, in addition to a supplement of remodeling annuloplasty. Neo-Chordae were used via a premeasured moveable loop technique [12] with ventricular placed knots. In forms of mitral regurgitation not associated with chordal rupture or elongation, correction of the MV was performed only by remodeling annuloplasty. The annuloplasty rings used were semi-rigid, complete rings (Carpentier Edwards Physio II^®^, Edwards Lifesciences Corp. or Sorin Memo 3D ReChord^®^, LivaNova PLC) size range 32–34 mm. In select cases when necessary, cleft sutures were added between valvular segments to adjust valve segment-height and optimised tight closure.

In the replacement group, the preservation of the attachment of the chordae tendineae and papillary muscles to the mitral valve annulus was achieced. The anterior leaflet of the mitral valve was divied through its midpoint and separated from the fibrous annulus and the posterior leaflet was retained. The interrupted sutures technique was used to attach a prosthetic valve to mitral annulus by using 2/0 Ethibond pledgeted sutures. The Prosthesis used were Bio-prosthesis (Carpentier-Edwards PERIMOUNT Plus mitral pericardial valve^®^, Model 6900P. Edwards Lifesciences Corp.) or mechanical prosthesis (Mitral Valve Standard Cuff, SJM™ Master Series Valves, Model MJ-501, Abbott) in sizes between 25 and 33 mm.

### Echocardiography

The diagnostic test for MVR was performed by echocardiography as a standard diagnostic test in the initial evaluation of patients with known or suspected valvular heart disease [4].

Transesophageal echocardiograms were performed intraoperatively in all patients after repair or replacement of the valve, whereas transthoracic echocardiograms (TTE) were performed routinely within 30 days and after 3 years in all patients after surgery, either in our institute or by the patient’s cardiologist.

### Statistical analysis

Categorical variables were presented as absolute numbers with percentages. Their distributions were compared between the groups with the chi-square test if its assumptions were met. Otherwise, we used Fisher’s exact test. Due to skewed distributions, all continuous variables were presented as median values with quartiles in brackets. We compared their distributions between the groups with the Mann–Whitney test. The survival data was collected through telephone follow-up and/or reports of the cardiologists, general practitioners, or reports from the resident registration office and survival analysis was made with the use of the Kaplan–Meier method. Using Cox regression models and calculating hazard ratios and 95% confidence intervals, analyses of data were conducted to identify risk factors for mortality in 30 days and 5 years. The survival functions of the analysed groups were compared with the log-rank test. Overall, *p*-values < 0.05 were considered statistically significant. For the statistical analysis, we used the R software v. 4.0.3. (Foundation for Statistical Computing, Vienna, Austria), as well as IBM SPSS v. 27.0.

### Study end-points

A primary end-point in this study was defined as all-cause mortality within 5 years, calculated from the date of surgery. Secondary end-points were recurrent MVR, re-operation, endocarditis and/or mortality within 30 days, 1, 3 and 5 years after surgery.

## Results

Two hundred forty one patients underwent an isolated mitral valve (MV) surgery in our center. Propensity score matching identified 128 patients, which were classified into two groups; the MV repair group (MVRe) and the MV replacement group (MVRp) with 64 patients in each group.

### Patients’ characteristics

The median age of patients undergoing MVRe was 70.50 (42–86) years, the median age of patients who underwent MVRp was 71.50 (48–88) years (*p* = 0.75). In our study, 30 patients in the repair group and 32 in the replacement group were females (46.9% and 50% respectively, *p* = 0.72). The median weight was 79.50 (54–115) kg in the MVRe group and 77.50 (47–138) kg in the MVRp group (*p* = 0.90).

Clinically 73.4% of patients in MVRe and 79.7% in MVRp were admitted with NYHA classification III/IV (*p* = 0.36). EuroSCORE II was used to predict the mortality risk in our population in a standard fashion [13]. The median EuroSCORE II in the MVRe group was 1.5% (0.56–14.4%) and 1.67% (0.5–25.43%) in the MVRp group (*p* = 0.2).

25 in the MV repair group and 24 in the MV replacement group (39.1% and 37.5% respectively, *p* = 0.86) had coronary heart disease not requiring surgery in their history. Previous myocardial infarction in patient history was observed in 3.1% of the repair group and 9.4% of the replacement group (*p* = 0.27). A history of atrial fibrillation (AF) has been documented in 46.9% of patients in the MVRe group and 51.6% in the MVRp group (*p* = 0.6). One patient in each group had previous cardiac surgery, 2 patients in the MVRe group and 7 in the MVRp group had at least one stroke in the patient history (*p* = 0.16).

Of the 22 patients with diabetes mellitus, 8 underwent MV repair and 14 underwent replacement (12.5% and 21.9% respectively, *p* = 0.16). 49 patients with obesity (BMI ≥ 30 kg/m^2^) were identified, 22 underwent MV repair and 27 underwent MV replacement (34.4% and 42.2% respectively, *p* = 0.36). Hypertension was observed in 105 patients, MV repair was performed in 51 (79.7%) patients and MV replacement in 54 (84.4%) patients (*p* = 0.49) ( Table 2).

**Table 2:** Patients Characteristics:

	Repair (N = 64)	Replacement (N = 64)	P value
Age (years)	70.5 (42–86)	71.5 (48–88)	0.75
Gender (n, %) female	30 (46.9%)	32 (50%)	0.72
Weight (kg)	79.5 (54–115)	77.5 (47–138)	0.90
Past history			
Coronary heart disease (n, %)	25 (29.1%)	24 (37.5%)	0.86
Atrial fibrillation (n, %)	30 (46.9%)	33 (51.6%)	0.60
COPD^a^/Asthma bronchial (n, %)	8 (12.5%)	16 (25%)	0.07
Myocardial infarction (n, %)	2 (3.1%)	6 (9.4%)	0.27
Previous cardiac surgery (n, %)	1 (1.6%)	1 (1.6%)	1.00
Stroke (n, %)	2 (3.1%)	7 (10.9%)	0.16
Ejection Fraction ≥ 50% (n, %)	46 (71.9%)	51 (79.7%)	0.43
NYHA^b^ classification			
I	6 (9.4%)	8 (12.5%)	0.36
II	11 (17.2%)	5 (7.8%)	
III	30 (46.9%)	36 (56.3%)	
IV	17 (26.5%)	15 (23.4%)	
Risk factors			
Diabetes mellitus (n, %)	8 (12.5%)	14 (21.9%)	0.16
Hypertension (n, %)	51 (79.7%)	54 (84.4%)	0.49
Obesity (n, %)	22 (34.4%)	27 (42.2%)	0.36
Smoking (n, %)	13 (20.3%)	22 (34.4%)	0.07
Hyperlipidaemia (n, %)	17 (26.5%)	24 (37.5%)	0.18

^a^COPD: chronic obstructive pulmonary disease

^b^NYHA: New York Heart Association

### Preoperative echocardiography

Preoperative echocardiography was performed as a standard diagnostic test for MVR. A normal left ventricular ejection fraction (LVEF ≥ 50%) was observed in 71.9% of patients in the repair group and 79.7% in the replacement group (*p* = 0.43). 31 (24.21%) patients underwent surgery with an impaired left ventricular function with an ejection fraction calculated at under 50%. A left atrial diameter ≤ 50 mm was observed in 64.1% in the repair group and 56.3% in the replacement group (*p* = 0.37). A left ventricular end-diastolic diameter ≤ 65 mm was observed in 89.1% of the repair group and 95.3% of the replacement group (*p* = 0.19).

The Carpentier classification (Cc) was used in this study to identify MV pathology. In our study, 26 of 128 (20.3%) patients had a Cc type I. Of these, 20 underwent MV repair and six underwent MV replacement (31.3% vs. 9.4% respectively, *p* = 0.002). Of the 67 (52.3%) patients who were admitted with Cc type II, 39 underwent MV repair versus 28 who underwent MV replacement (60.9% vs. 43.6% respectively, *p* = 0.05). Of the 28 (21.9%) patients who presented with Cc type IIIa, two underwent MV repair and 26 underwent MV replacement (3.1% vs. 40.6%, *p* < 0.001). Of the seven (5.5%) patients admitted with functional MVR (Cc type IIIb), three underwent MV repair and four underwent MV replacement (4.7% vs. 6.3% respectively, *p* = 1.0).

### Intraoperative findings

In our study population, the indication for surgery was elective in 93 of 128 (72.7%) patients, whereas surgery was urgent in 27 (21.1%) and emergent in 8 (6.3%). The aortic cross-clamp time (X-clamp time), cardiopulmonary bypass time, and duration of surgery were not statistically significant in both groups (Table 3).

**Table 3:** Intraoperative findings

Intraoperative findings	Repair (N = 64)	Replacement (N = 64)	*p* value
Indication of surgery			
Elective (n, %)	50 (78.1%)	43 (67.2%)	0.17
Urgent (n, %)	14 (21.9%)	13 (20.3%)	0.83
Emergency (n, %)	0 (0%)	8 (12.5%)	0.006
CPB^a^ time (min.)	90.5 (56–175)	99.5 (50–265)	0.10
X-clamp time^b^ (min)	58 (33–122)	66 (36–171)	0.13
Duration of surgery (min.)	166.5 (107–275)	178 (100–387)	0.41

^a^CPB: Cardiopulmonary bypass

^b^X-Clamp time: aortic cross-clamp time

### 30-day outcome

A reoperative procedure within 30 days was performed on two patients after MV repair with a diagnosis of systolic anterior motion (SAM) of the mitral valve and chordae tendineae rupture. Both underwent a valve replacement on the 1st and 6th postoperative day respectively. No reoperation was performed in the replacement group (*p* = 0.5). We observed no major adverse cerebrovascular events and a single case of mesenteric ischemia in both groups.

Operative mortality was defined as death within 30 days after surgery or during the same hospitalisation [14, 15]. In our study, we observed no significant difference in 30-day mortality rates between the comparing groups. Two patients in the MV repair group and 5 patients in the MV replacement group (3.1% vs. 7.8%, *p* = 0.44) passed away in the early hospital stay. Both patients in the repair group died due to postoperative cardiogenic shock and in the replacement group, one patient died with septic shock, one patient with mesenteric ischemia and the other patients demised with cardiogenic shock.

### Long-term follow-up

During the follow up period, we collected echocardiographic findings, performed on our study population by cardiologists upto 9 years for the complete follow-up period. We have achieved a complete follow up for the entire investigated patient cohort. They demonstrated 4 patients in the MVRe group and no patient in the MVRp group had severe MV regurgitation (2 patients with sole regurgitation and 2 patients with annular ring dislocation) 3 years after surgery (*p* = 0.09). A mitral valve replacement was performed on one patient and the other 3 patients underwent mitral valve re-repair; one patient is currently listed for heart transplant due to preoperatively known cardiomyopathy.

We observed altogether eight patients who required redo surgery within the follow-up period, 2 patients in MVRp and 6 in MVRe group (3.8% vs. 10.2% respectively, *p* = 0.28) (Table 4). A mitral valve replacement was performed in five cases. Only one patient developed endocarditis after 6 months of MV replacement. This was treated operatively with a re-replacement.

**Table 4:** Redo surgery

Primary surgery	Cause of redo	Secondary surgery	Time of redo after
MVRp^a^	Endocarditis	Replacement	6 Months
	paravalvular leak	Replacement	2 Months
MVRe^b^	chordal rupture	Replacement	6 days
	SAM^c^ Phenomena	Replacement	1 day
	Severe regurgitation	Replacement	3.5 years
		Repair	2 years
	Annular ring dehiscence	Repair	3 Months
		Repair	2.5 years

^a^MVRp: mitral valve replacement

^b^MVRe: mitral valve repair

^c^SAM: systolic anterior motion

**Fig. 2:**
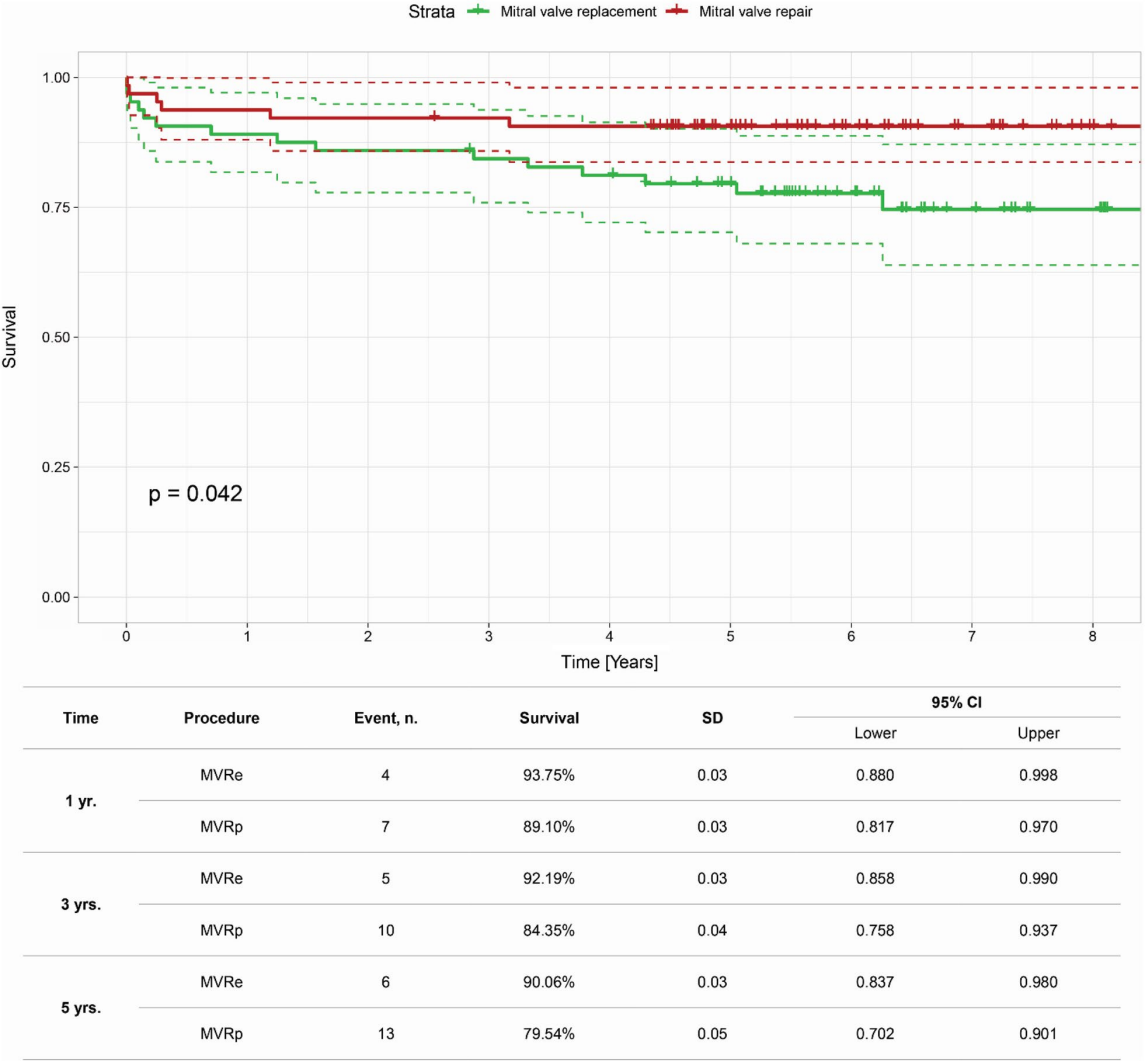
Kaplan–Meier

Primary end-point showed overall survival of the total population after 5 years was 85.1%. Survival in the repair group was 90.06% after 5 years and 79.54% in the replacement group (*p* = 0.04) (Fig. 2).

### Predictors of short- and long-term mortality

**Fig. 3:**
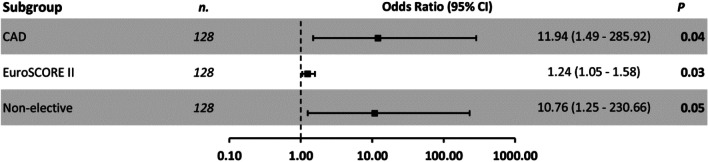
Multivariate logistic regression analysis for 30 day-mortality

As determined by the multivariate logistic regression model, patients with higher EuroSCORE II (1.24: OR 95% CI 1.05–1.5, *p* = 0.03), non-elective surgery (10.76: OR 95% CI 1.25–230.66, *p* = 0.05) or coronary artery disease (11.94: OR 95% CI 1.49–285.92, *p* = 0.04) were at high risk for 30 day-mortality (Fig. 3).

**Fig. 4:**
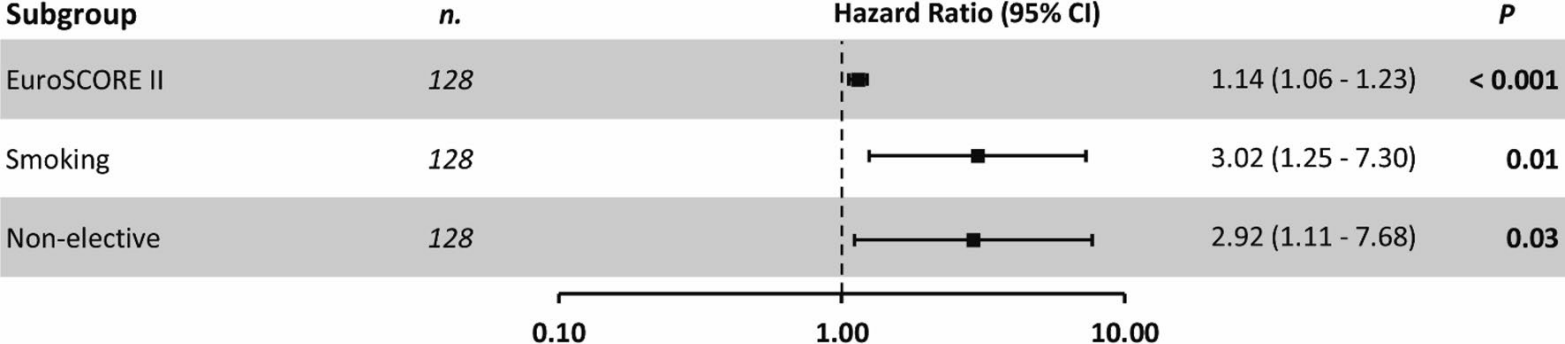
Multivariate Cox regression analysis for 5 year-mortality

**Fig. 5:**
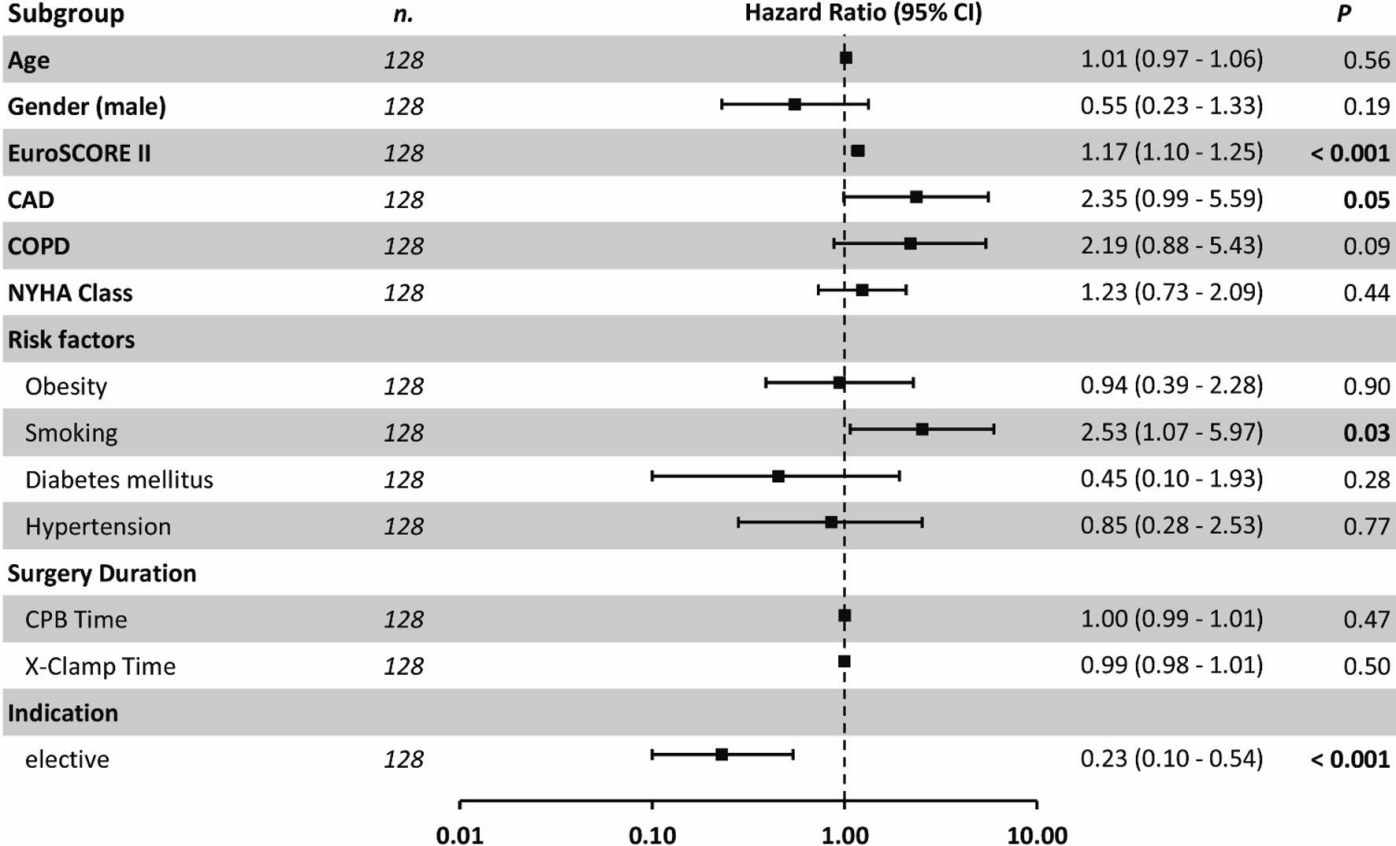
Univariate Cox regression analysis for 5 year-mortality

According to the multivariate cox regression model we observed higher EuroSCORE II (1.14: HR 95% CI 1.06–1.23, *p* < 0.001), non-elective surgery (2.92: HR 95% CI 1.11–7.68, *p* = 0.03) or Smoking (3.02: HR 95% CI 1.25–7.30, *p* = 0.01) to be associated with a high risk of 5-year mortality (Figs. 4, 5).

## Discussion

The optimal surgical treatment for MVR remains under debate. The latest reports and study presentations demonstrate mitral valve repair is considered to be superior to replacement [7, 16]. The excellent outcomes of surgical repair with a recommendation of risk stratification and earlier intervention when the probability of durable repair is high show low operative mortality and morbidity rates [2, 4, 7]. In view of these findings, the rate of MV repair in Germany increased from 37.6 to 62.8% between 2000 and 2015 [17]. These findings however remain debatable considering reports showing a benefit for preventing recurrence of mitral valve regurgitation and rehospitalisation with mitral valve replacement. We decided to analyse our institutional results comparing mitral valve repair and mitral valve replacement using propensity-matched analysis to homogenize the treatment groups and to perform a detailed statistical comparative analysis.

The age of a patient has long been considered an independent predictor of MV replacement [18]. Silaschi et al. [19] showed patients who underwent MV repair were older compared to the MV replacement group. Thourani et al. [20] found that survival in the MV repair group was significantly higher than that in the MV replacement group in patients younger than 60 years, whereas this difference was not visible in patients older than 60 years. In our study, propensity score matching was performed prior to analyse, with age being one of the baseline variables for matching (Tables 1, 2, 3 and 4).

Vassileva et al. [18] demonstrated that patients who had diabetes mellitus, myocardial infarction, stroke, previous cardiac surgery, and/or previous percutaneous coronary intervention in their history had a tendency to undergo MV replacement. In our study, the forementioned characteristics as well as the EuroSCORE II were similar between the two groups. In light of this, we believe that the decision against MV repair should not be based on the patient’s age or history.

It is known that many factors are affecting the short and long-term outcomes of mitral valve surgery. In our study, by performing a univariate logistic and multivariate regression analysis, we found that patients with coronary artery disease, or a history of it, had increased risk of in-hospital mortality. That could be due to reduced heart function as a result of the disease, and the acute side effects of cardiopulmonary bypass and cardioplegia which may cause an impairment in heart function in the first few hours after surgery, thus increasing the probability of developing low-cardiac output syndrome and the respective impact. Similar to Carino et al. [13], our study showed that a high EuroSCORE II was associated with an increased 30-day mortality rate and higher adverse long-term outcomes. EuroSCORE II is widely considered an important predictor for 30-day mortality after cardiac surgery. It was validated in some studies in patients undergoing mitral valve surgery [13, 21, 22] but we only found one study that explored the ability of EuroSCORE II to predict the 30-day mortality in patients undergoing mitral valve surgery. They found that EuroSCORE II overestimates 30-day mortality [13].

Some surgeons believe MV repair to be more complex, requiring longer X-clamp time and having a higher risk of recurrence [19]. In our analysis, we observed that the difference between the two groups in cardiopulmonary bypass and X-clamp time were not statistically significant. These results were in agreement with the findings of Silaschi et al. [19] and Chivasso et al. [23], In contrast, Farid et al. [24] showed that patients who underwent MV repair to have had shorter cardiopulmonary bypass and X-clamp times compared with those who underwent MV replacement. We know that cardiac and operative trauma can be reduced when cardiopulmonary bypass and X-clamp time are shorter, depending on the surgeon’s experience.

In this study, we observed two cases of ring dehiscence requiring re-surgery. This complication after atrioventricular valves repair, particularly after mitral valve repair, is not rare and is reported in 13–42% of procedural failures in mitral valve annuloplasty repair [25]. This complication led us to implement a modified approach by placing four pledgeted sutures on the A1, A3, P1 and P3 segments to reinforce the stability of the ring. Since using this strategy, we have not observed any new cases of ring dehiscence in our cohort, as well as in patients not included in this trial.

The American Association for Thoracic Surgery Guidelines, American Heart Association/American College of Cardiology and the European Society of Cardiology recommend MV repair to treat severe primary MVR [2, 4, 26]. The evidence for this recommendation is derived from single-center studies; however, the superiority of MV repair led the guidelines to consider the probability of achieving a durable repair in specialized MV repair centers [2, 4]. Some retrospective studies have reported advantage of MV repair, in particular the operative mortality being lower compared to that of MV replacement [27–30]. However, there are a multitude of studies showing no evidence for a preference of one intervention over the other in patients with endocarditis, secondary MVR or mitral valve surgery in combination with coronary artery bypass grafting [10, 16, 20, 28, 31].

This clinical investigation demonstrates that patients undergoing MV repair to have similar mortality rates within 30 days after surgery, but lower mortality rates after 5 years compared with patients undergoing MV replacement.

## Strengths

The strong point of this study was that surgery was mainly performed by two surgeons following the same surgical strategy and techniques, reducing the operative bias. The strict implementation of rigid inclusion/exclusion criteria allowed for strong propensity matching with similar preoperative patients’ characteristics in both groups, leading to the credibility of short and long-term results. Furthermore, all patients, included in this study after propensity-score matching, were followed up.

## Limitations

The limitations of this study include the single-center nonrandomized retrospective study design and the limited number of patients. These may weaken the conclusions of the study.

## Conclusions

In patients with moderate to severe mitral valve regurgitation, MV repair can be performed with lower operative and 5-year mortality rates and is associated with better postoperative outcomes. Decision making for MV replacement vs. repair is influenced by the pathology of the MV. We agree that MV repair should be the treatment of choice for severe mitral regurgitation and should remain a central aspect in treatment algorithms and the quality measures of valve centers.

## Data Availability

The datasets used and/or analysed during the current study are available from the corresponding author on reasonable request.

## Abbreviations

Cc: Carpentier classification
COPD: Chronic obstructive pulmonary disease
CPB: Cardiopulmonary bypass
LVEF: Left ventricular ejection fraction
MVR: Mitral valve regurgitation
MVRe: Mitral valve repair
MVRp: Mitral valve replacement
NYHA: New York Heart Association
SAM: Systolic anterior motion
STEMI: ST-segment elevation myocardial infarction
TTE: Transthoracic echocardiogram
X-clamp time: Aortic cross-clamp time
